# Does Sensory Integration Influence Gait Parameters in Healthy Older Adults? Insights from a Systematic Review with Meta-Analysis

**DOI:** 10.3390/jcm14134545

**Published:** 2025-06-26

**Authors:** Esma Nur Kolbaşı, Elisabeth G. van der Hulst, Joke Spildooren, Lotte Janssens, Pieter Meyns

**Affiliations:** 1 REVAL Rehabilitation Research Center, Hasselt University, 3590 Diepenbeek, Belgium; esma.kolbasidogan@uhasselt.be (E.N.K.); liset.vanderhulst@uhasselt.be (E.G.v.d.H.); joke.spildooren@uhasselt.be (J.S.); lotte.janssens@uhasselt.be (L.J.); 2 Department of Nutrition and Movement Sciences, NUTRIM Institute of Nutrition and Translational Research in Metabolism, Maastricht University, 6229 ER Maastricht, The Netherlands

**Keywords:** aged, sensory interaction, sensory orientation, postural balance, postural control

## Abstract

**Background/Objective:** Sensory integration (SI) involves the central processing of visual, vestibular, and somatosensory inputs. It plays a key role in regulating movements such as gait. However, aging may impair these systems and SI, altering the gait. Therefore, this systematic review and meta-analysis aim to examine the relationship between gait parameters and SI during standing in healthy older adults. **Methods:** A systematic literature search was conducted in the Web of Science, PubMed, MEDLINE, and PEDro databases. Correlation coefficients between gait speed, sway (area and/or velocity) while standing under different SI conditions, and quotients were extracted. The Romberg Quotient (RQ) and Proprioception Quotient (PQ) were used to assess reliance on visual and somatosensory systems, respectively. The studies were grouped by condition, quotient, and outcome measures for the meta-analysis. **Results:** Thirteen studies (*n* = 719, mean age 72.5 years) were included. There were significant associations between gait speed and sway area during standing with eyes open on a stable surface (r: −0.235, *p* < 0.001), eyes closed on a stable surface (ECS) (r: −0.201, *p* < 0.001), eyes open on a compliant surface (r: −0.198, *p* < 0.001), and eyes closed on a compliant surface (r: −0.186, *p* < 0.004). No associations were found between gait speed and sway velocity in each condition, RQ, and PQ (*p* > 0.486 for all), except for the ECS (r: −0.149, *p*: 0.01). **Conclusions:** This study indicated a partial link between gait speed and SI in older adults. Future research should focus on dynamic SI evaluation to better understand this association.

## 1. Introduction

Walking is an important activity in our daily lives. Walking may seem effortless even though the execution of gait is an intricate process requiring harmonious cooperation between the sensory and motor systems. Sensory information regarding the task and the environment, gathered through the visual, somatosensory, and vestibular systems, plays a crucial role in modulating movement [1,2]. The somatosensory system provides input from the surface characteristics via proprioceptors and cutaneous receptors while the orientation of the head in space and the information regarding gravity are provided by the vestibular system [3,4]. The visual system provides cues on the surrounding environment [5]. These afferent inputs, when available, are subsequently processed in the cortices, a mechanism known as “*sensory integration (SI)*”. Based on this integrated sensory information, an efferent output response can be generated, which may ultimately manifest as the movement of the different body segments and, in turn, can lead to walking [1,5].

In younger adults, when there is a decrease in the (reliability of) information provided by one of the sensory systems, the central nervous system optimally reweights the sensory systems to compensate for the insufficiency of that system to maintain task performance (i.e., gait) [6,7,8]. However, with an advancing age, a decline in sensory, motor, and central processing may occur [9,10], which, in turn, leads to gait changes and increased fall risk in older adults [11,12,13,14]. For instance, aging affects both peripheral and central vestibular structures [15,16,17,18]. These impairments are closely linked with spatiotemporal gait changes in older adults including increased stride length and stance time [19]. Similarly, in a study, when older adults were asked to walk on a compliant surface, they exhibited a lower walking speed with pronounced hip and knee flexion compared to walking on a firm surface, suggesting a somatosensory decline [20]. Furthermore, age-related decline in visual contrast sensitivity was found to be associated with lower gait speed and stride length in older adults [21].

Importantly, research also indicates that sensory reweighting, the dynamic process by which the nervous system adjusts the relative contribution of different sensory inputs, is often slowed or diminished in older adults [22,23]. Some studies reported that older adults were more reliant on their visual systems [24] whereas others highlighted increased dependence on the somatosensory system [25]. Nonetheless, the age-related decline in sensory reweighting in older adults may lead to compromised motor control, contribute to altered gait patterns, and increase the risk of falls [26,27,28].

To evaluate these complex SI processes, stabilometry measurements with alternating sensory conditions (e.g., eyes open, eyes closed) and SI tests (e.g., the Sensory Organization Test (SOT)) are commonly used in older adults. While one’s reliance on their visual, vestibular, and somatosensory systems can be individually evaluated within the framework of the SOT, stabilometric measurements require the calculation of specific quotients [29,30,31]. Such quotients allow us to quantify and interpret whether a person relies more on a specific sensory system to maintain balance. Specifically, the Romberg Quotient (RQ) and Proprioception Quotient (PQ) are established measures of reliance on visual and somatosensory systems, respectively [31,32].

Although numerous studies have suggested a potential link between sensory system changes and gait parameters in older adults, the findings remain inconsistent, and the extent to which individual sensory modalities and sensory reweighting strategies specifically contribute to gait performance has not yet been clearly established [33,34,35,36,37]. Accordingly, our aim with this systematic review and meta-analysis was to investigate the association between gait parameters and the SI process, encompassing both individual sensory conditions and sensory reweighting mechanisms, in healthy older adults. To the best of our knowledge, no comprehensive review and/or meta-analysis had been conducted to examine this association. We hypothesized that in older adults, (i) alterations in gait parameters would be linked to greater postural sways under individual sensory challenging conditions and (ii) deviations in specific gait parameters would be associated with suboptimal sensory reweighting during postural control, such as via a dominant reliance on the visual system.

## 2. Materials and Methods

This review was reported based on the Preferred Reporting Items for Systematic Reviews and Meta-Analysis (PRISMA) guidelines [38]. The study was registered in PROSPERO with registration number CRD42024527627.

### 2.1. Search Strategy

A systematic literature search was conducted in the PEDro, PubMed, MEDLINE, and Web of Science databases in January 2025. Only a language filter was applied, and articles in English were listed. The search strategy and the P.I.C.O. are presented in Supplementary Material S1.

### 2.2. Study Selection Criteria

Studies were considered eligible if they met the following criteria: (a) published as a full text in English; (b) including community-dwelling healthy older adults (>60 years for each participant); (c) including quantitative, continuous measures of SI, assessed in bipedal stance, with at least one sense (visual, somatosensory, or vestibular) being perturbed for testing; and (d) including quantitative, continuous parameters of gait (i.e., spatiotemporal parameters and/or kinematics and/or kinetics). Exclusion criteria were as follows: (a) studies including participants with known pathologies such as malignancy, stroke, Parkinson’s disease, neuropathy, vestibular disorders, uncorrected visual problems, etc. and (b) studies with only clinical SI and/or gait assessment methods (e.g., only classification scoring). We have also not included case reports or case series. The authors of the original studies were contacted via email to clarify any uncertainties regarding eligibility criteria.

Duplications in the databases were detected with Mendeley Reference Manager (Version 1.19.8- Elsevier, London, UK). The studies were screened for title and abstract eligibility. Then, a full-text screening was carried out by two authors (E.N.K. and E.G.v.H.). Any disagreement between these two authors was resolved by the third author (P.M.).

### 2.3. Quality Assessment

The risk of bias was evaluated with a modified version of the Quality in Prognosis Studies (QUIPS) tool [39]. The modifications were made as described in the previous study [40]. For instance, items on drop-outs were omitted since the information necessary for this study was cross-sectional in nature and did not require follow-ups. The QUIPS tool assesses the risk of bias in six domains: study participation, study attrition, prognostic factor measurement, outcome measurement, confounding factors, and statistical analysis and reporting. All domains are rated as having low, moderate, or high risk of bias. Correlation coefficients obtained through direct contact with the authors were considered to have a high risk of bias, as these data had not undergone peer review [41].

Quality assessment was separately conducted by two authors (E.N.K. and E.G.v.H.). Any disagreement was resolved through a discussion with the third author (L.J.).

### 2.4. Data Extraction

Firstly, we contacted authors for the studies that did not report correlation coefficients between gait parameters and each SI condition, RQ, and/or PQ. The RQ and PQ were included in this meta-analysis to evaluate one’s reliance on their sensory systems as they are established metrics for assessing sensory reliance [31,32]. The RQ represents one’s reliance on their visual system to maintain their balance [32]. Similar to the RQ, the PQ represents a reliance on the somatosensory system for balance control [31]. RQ and PQ were calculated as [31] follows:

$$RQ=\frac{\left(Eyes\;Closed,\;firm\;surface\right)}{\left(Eyes\;Open,\;firm\;surface\right)}\;PQ=\frac{\left(Eyes\;open,\;compliant\;surface\right)}{\left(Eyes\;open,\;firm\;surface\right)}$$

Authors were emailed at least three times for the data sharing. If they provided either raw data or the correlation coefficients, the study in question was included in the systematic review and meta-analysis.

Then, one author extracted the data (E.N.K.) and another author (E.G.v.H.) checked and confirmed the accuracy of the extraction. The following information was extracted from each of the studies: (a) study details (first authors, year, sample size), (b) demographics (age, gender, body mass index (BMI), previous falls), (c) experiment-specific details (SI test, conditions, foot position, and gait test), (d) type(s) of outcome measurement(s) for the prognostic factor (i.e., 95% ellipse area), and (e) findings (correlation coefficients and *p*-values).

### 2.5. Data Synthesis and Analysis

The extracted data were grouped first based on the condition, RQ, and PQ. Then, studies were further grouped based on the reported outcome measurements. An outcome measure had to be present in at least three articles to proceed with a meta-analysis [40,42]. Outcome measures were then clustered as sway area (95% ellipse area, trunk sway area, and trace length) and sway velocity (sway velocity of the trunk, mean CoP velocity, and Sway Velocity Index). The same principle was applied to RQ and PQ as well [43]. Other stabilometric variables, such as frequency-domain metrics, were not included as they had not been reported in any of the eligible studies. A list of the meta-analyses conducted is presented in Table 1.

The meta-analyses were conducted in IBM SPSS Statistics version 29.0 (IBM SPSS Statistics, Armonk, NY, USA), using correlation coefficients, without differentiating between Spearman and Pearson correlation coefficients [44]. If one study reported more than one correlation coefficient, such as in different directions (i.e., anteroposterior or mediolateral directions), the mean value of these correlation coefficients was calculated and recorded to represent a single effect size for that condition [45]. This approach was chosen to ensure methodological consistency across the meta-analysis. If a study reported separate correlation coefficients for different tasks (e.g., normal and narrow stance width), these correlation coefficients were included as distinct datasets in the meta-analyses. Later, all correlation coefficients were transformed to Fisher’s Z value [46]. Additionally, the variance of Fisher’s Z-transformed correlations was calculated as suggested by Hedges [47]. Next, meta-analyses were conducted using a random-effects model, and an inverse Fisher’s z-transformation was applied to determine the pooled correlation coefficient and its 95% confidence interval (95% CI) [48]. Pooled correlation coefficients were interpreted as follows: (a) small if the r < 0.30, (b) moderate if 0.30 ≤ r < 0.50, and (c) strong if r ≥ 0.50 [49].

The I^2^ test was used to evaluate heterogeneity among the studies [50]. It was interpreted as showing negligible heterogeneity if the I^2^ < 40% [50]. Moderation and sensitivity analyses were planned to be conducted if the I^2^ value was above 40% to identify the sources of this heterogeneity [48]. At least four studies were required to perform moderation and/or sensitivity analyses [51].

## 3. Results

### 3.1. Study Selection

**Figure 1:**
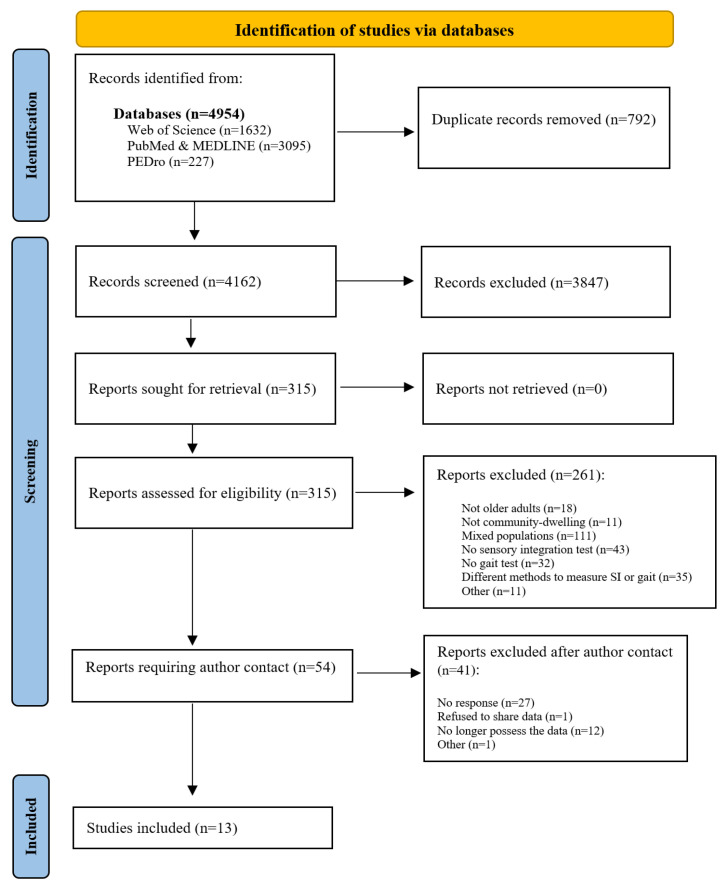
PRISMA flowchart.

This systematic review included 13 studies ranging from 1996 to 2024. A summary of the literature search is provided in Figure 1. Among the 13 studies reviewed, we were unable to establish contact with the authors of three studies. Since these studies already reported some correlation data, we could include them in the systematic review and meta-analysis.

### 3.2. Characteristics of the Participants

A total of 719 participants (mean age 72.5 years, 67.7% female) were included in the studies. The ages of the participants ranged from 60 to 102 years. However, eight studies did not report their age ranges [33,37,52,53,54,55,56,57]. The mean BMI of the participants was 32.1 kg/m^2^. However, the BMI was not reported in seven studies [34,52,53,55,57,58,59]. Four studies included participants who had experienced single and/or multiple falls in the past year, along with the non-fallers [34,53,54,57]. Further details of participant characteristics are presented in Table 2.

### 3.3. Outcome Measurements

Three studies used the SOT [34,55,59], and one study [56] used the Modified Clinical Test of Sensory Interaction and Balance (mCTSIB) to measure SI, whereas the remaining nine studies used postural control measurements with sensory perturbations (i.e., without standard SI tests) [33,37,52,53,54,57,58,60,61]. The eligible studies included in this systematic review and meta-analysis, except for two [37,60], exclusively evaluated gait speed, and the use of other gait parameters was sparse. Espinoza-Araneda et al. [60] reported foot clearance height (mm), cycle duration (s), and stride length (m) in addition to gait speed (see Table 2). Si et al. [37] reported an extensive list of gait parameters; however, not all of these parameters were suitable to be included in this systematic review. Therefore, we have only included gait speed and cadence parameters from Si et al. [37]’s study. Six studies used standardized walking tests for predefined distances such as the 10 m Walk Test to evaluate gait speed [33,52,54,58,59,61] whereas two studies used walking for predefined time such as the 6 min Walk Test [55,56]. On the other hand, five studies used unstandardized tests to define gait speed [34,37,53,57,60]. Further details of the study protocols and participant characteristics are presented in Table 2.

### 3.4. Risk of Bias

All included studies had a high risk of bias as presented in Table 3. Ten studies had a high risk of bias, mainly due to study confounding and statistical analysis, and reporting domains of the QUIPS [52,53,54,58,59,60,61]. Since the correlation coefficients in these studies were obtained through author contact and have not undergone a peer-review process, the aforementioned parts were scored as having a high risk of bias, which ultimately led to the overall scoring of a high risk of bias. Two studies had a high risk of bias due to several domains, namely study participation, attrition, outcome measurement, study confounding, and statistical analysis and reporting [33,34]. The remaining study had a high risk of bias due to the study confounding domain even though the correlations were not obtained through direct author contact [58].

### 3.5. Qualitative Synthesis of the Findings

As presented in Table 2, three studies used the *equilibrium score* as an outcome measure to report SI [34,55,59]. Since the conditions assessed in these studies did not overlap [34,55,59], a meta-analysis could not be conducted. Two of the studies reported a non-significant correlation between gait speed and equilibrium score while standing with eyes open on a stable surface (EOS), EOS with sway-referenced visual surroundings, eyes open on a compliant surface (EOC), and eyes closed on a compliant surface (ECC) [34,55] while the third study did not report on these correlations [59]. These studies did not detect a relationship between gait speed and quotients [55,59]. The third study investigated only the association between gait speed and vestibular quotient (ECC/EOS); however, it did not find a significant relationship (r: 0.136/, *p*: 0.366) [34]. Collectively, these findings may suggest a non-significant association between gait speed and SI conditions or quotients when reported as an equilibrium score.

Also, two studies included gait parameters other than gait speed [37,60]. The findings of Espinoza-Araneda et al. [60] suggest that foot clearance and cycle duration were associated with sway areas in some of the SI conditions (see Table 2). However, there was no relationship between stride length and sway area in EOS (r: −0.008, *p*: 0.947), ECS (r: 0.040, *p*: 0.743), EOC (r: 0.132, *p*: 0.271), or ECC (r: 0.061, *p*: 0.613). None of the gait parameters were associated with the RQ and PQ. Si et al. [37] reported correlations between cadence and sway area and velocity in SI conditions (Table 2). It was found that cadence was associated with both sway area or velocity in ECS, in both foot positions (i.e., self-selected and narrow) (*p* < 0.039 for all).

### 3.6. Quantitative Synthesis of the Findings

**Figure 2:**
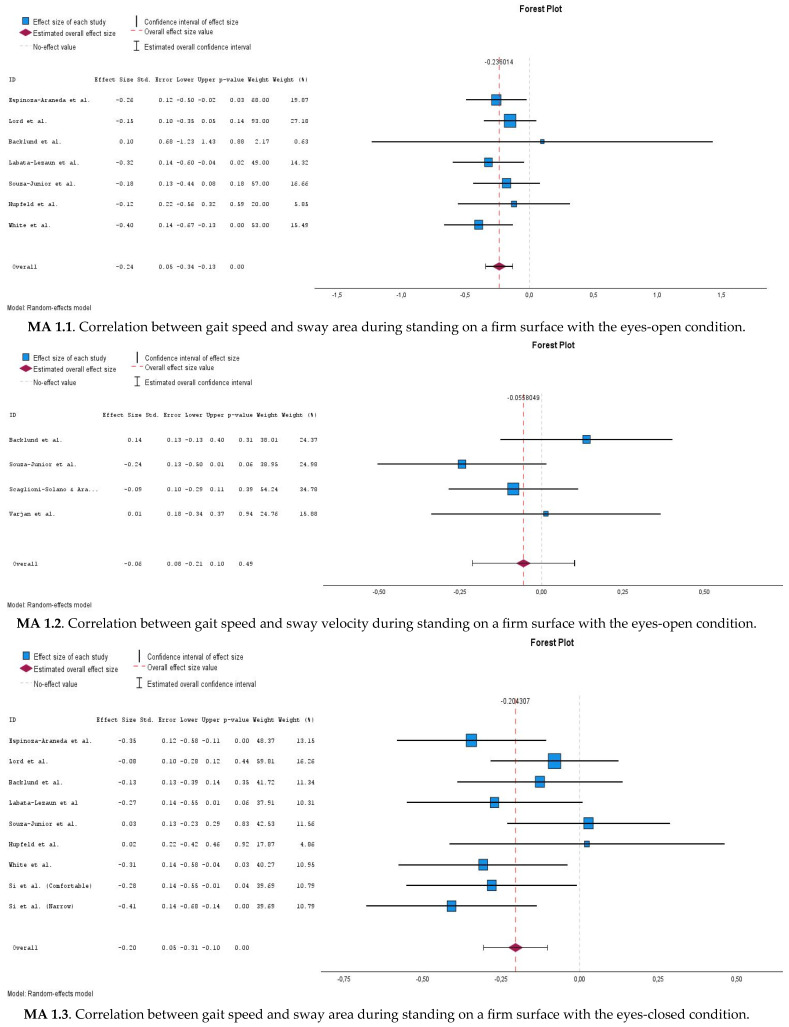

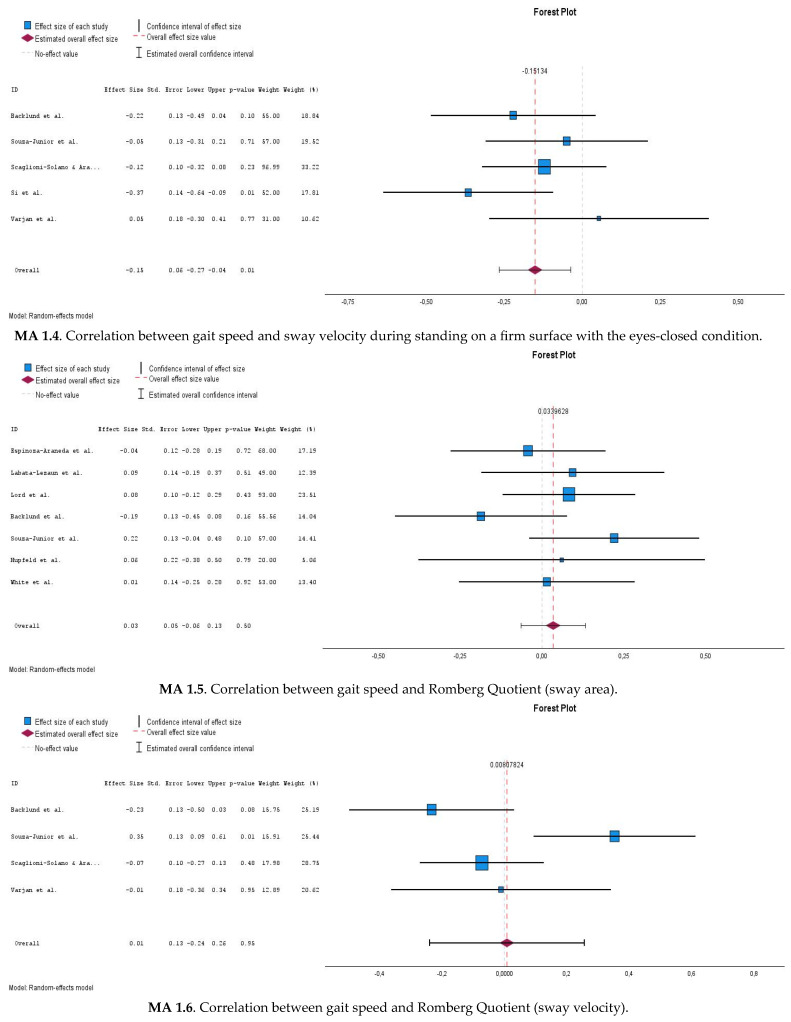

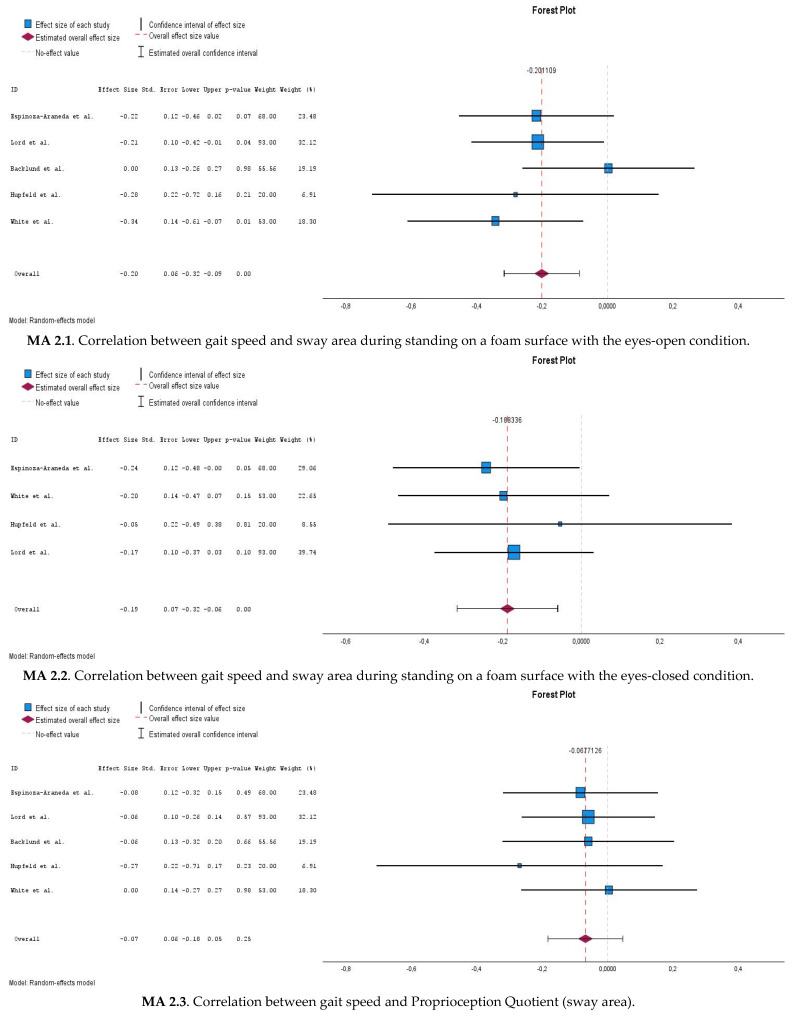
Forest plots of the meta-analysis in the study [33,34,37,52,53,54,55,56,57,58,59,60,61].

Table 4 and Figure 2 show the details of the conducted meta-analyses and the forest plots.

#### 3.6.1. Correlations Between Gait Speed and Postural Sway Measures During Eyes-Open Stable Surface Condition (MA 1.1 and MA 1.2)

Regarding the EOS condition, the meta-analyses of the relationships between gait speed and sway area (MA1.1) and sway velocity (MA1.2), seven studies (*n* = 416) [33,52,53,56,57,60,61] and four studies (*n* = 252) [33,52,54,58] were included, respectively. Even though a negative correlation (significant pooled effect size of −0.235 (95%CI: −0.340–(−0.13))) was observed for gait speed and sway area (MA1.1), a non-significant pooled correlation coefficient of –0.056 (95%CI: −0.213–0.101) was found for the relationship between gait speed and sway velocity (MA 1.2).

#### 3.6.2. Correlations Between Gait Speed and Postural Sway Measures During Eyes-Closed Stable Surface Condition (MA 1.3 and MA 1.4)

For the ECS condition, the meta-analyses of the associations between gait speed and sway area (MA1.3) and sway velocity (MA1.4) included nine datasets from eight studies (*n* = 526) [33,37,52,53,56,57,60,61] and five studies (*n* = 307) [33,37,52,54,58], respectively. A significant pooled effect size of −0.201 (95%CI: −0.306–(−0.102)) was observed for gait speed and sway area (MA 1.3) whereas MA 1.4 also produced a significant pooled effect size of −0.149 (95%CI: −0.266–(−0.037)) for the association between gait speed and sway velocity.

#### 3.6.3. Correlations Between Gait Speed and Romberg Quotient (MA 1.5 and MA 1.6)

For the RQ, seven (*n* = 416) [33,52,53,56,57,60,61] and four studies (*n* = 252) [33,52,54,58] were included in the meta-analyses of the associations between gait speed and sway area (MA1.5) and sway velocity (MA1.6), respectively. Non-significant pooled effect sizes of 0.034 (95%CI: −0.065–0.133) and 0.008 (95% CI: −0.240–0.256) were found in MA 1.5 for the association between gait speed and the RQ, measured as sway area, and in MA 1.6 for the relationship between gait speed and the RQ, assessed as sway velocity, respectively.

#### 3.6.4. Correlations Between Gait Speed and Postural Sway Measures During Eyes-Open Compliant Surface Condition (MA 2.1)

Regarding the EOC condition, the meta-analysis of the relationship between gait speed and sway area (MA2.1) included five studies (*n* = 304) [52,53,56,57,60]. A significant pooled correlation coefficient (pooled r = −0.198, 95% CI: −0.316–(−0.086)) was found between gait speed and sway area in EOC.

#### 3.6.5. Correlations Between Gait Speed and Postural Sway Measures During Eyes-Closed Compliant Surface Condition (MA 2.2)

For the ECC condition, four studies (*n* = 246) [53,56,57,60] were included in the meta-analysis for the relationship between gait speed and sway area (MA 2.2). A significant pooled correlation coefficient (pooled r = −0.186, 95% CI: −0.316–(−0.060)) was found between gait speed and sway area in ECC.

#### 3.6.6. Correlations Between Gait Speed and Proprioception Quotient (MA 2.3)

MA 2.3 also yielded a non-significant pooled effect size of −0.068 (95%CI: −0.183–0.047) between gait speed and PQ (assessed as sway area) (five studies, *n* = 304) [52,53,56,57,60].

### 3.7. Moderation and Sensitivity Analysis

One meta-analysis (MA 1.6) exhibited statistical heterogeneity exceeding 40%, prompting us to plan a meta-regression analysis. Given the noticeable differences in gender distribution across studies included in MA 1.6, the female percentage was examined as a moderator. This choice was theoretically justified as gender differences have been reported to influence gait, body sway, and the relationship between them [54,60,62]. The analysis confirmed its relevance, explaining 94% of the variance and eliminating heterogeneity (remaining I^2^: 13.9%). The findings are presented in Supplementary Material S2.

## 4. Discussion

This was the first systematic review and meta-analysis to explore the association between gait parameters and SI in healthy older adults, and the findings partially supported our initial hypotheses. Our first hypothesis concerning the link between gait parameters and postural sway under sensory challenging conditions was confirmed. We observed a significant negative association between gait speed and sway area during EOS, ECS, EOC, and ECC. These indicate that the higher the gait speed is, the lower the postural sway during SI conditions will be. However, our second hypothesis regarding the link between gait parameters and the RQ and PQ was not supported. In other words, healthy older adults’ reliance on vision and proprioception during standing was not associated with gait speed. Taken together, these findings suggest a partial link between gait speed and the SI process.

The majority of the studies in this systematic review only investigated gait speed, leading us to conduct a synthesis focused specifically on gait speed. This exclusive focus on gait speed in the current study was particularly relevant given its well-established relationship with fall risk in older adults [63,64,65]. This focus allows for a clearer understanding of how SI interacts with gait speed. More specifically, our findings revealed a negative association between gait speed and postural sway area under sensory challenging conditions in older adults. This result indicates that the higher the sway area in standing is, the lower the gait speed becomes, which may suggest an increased likelihood of falls [66,67,68]. However, these associations were very small as evidenced by the effect sizes (Table 4). These results are consistent with previous research findings. Zhou et al. [69] reported a significant, yet small (r:0.203), association between gait speed and the composite score (i.e., weighted average sway of all equilibrium scores in the six conditions) of the SOT in older adults. This finding suggests that gait speed is not solely dependent on SI capacity. This notion could be supported by the findings of Aranda-Garcia et al. [36] and Chung et al. [70], which demonstrated that gait speed was associated not only with body sway but also with lower extremity muscle strength. Thus, it may be rational to assume that gait speed may rely on other functions such as muscle strength [71].

Interestingly, the correlations between gait speed and sway area were not stronger when increasing the sensory challenge (i.e., the association did not progressively increase). Means et al. [72] also reported a similar relationship between the gait subscale of the Tinetti Index and the sway area under EOS and ECS. These findings may be explained by different compensatory mechanisms that could be at play. The correlation coefficient between gait speed and EOS in the current study and Means et al. [72]’s study was higher compared to the other conditions. This was probably due to the fact that EOS involves the recruitment of optimal proprioceptive and visual information. Furthermore, more challenging conditions such as ECS, EOC, and ECC may require compensatory mechanisms that vary across older adults as individuals adopt a unique strategy to cope with the task requirements based on their SI capabilities [73]. For instance, in the ECS condition, one older adult with a mild visual decline could reweight the importance of inputs from the other sensory systems and maintain the task successfully whereas another older adult with sensory or vestibular decline could be unable to do so, resulting in a worse performance than the former. Thus, in turn, the difference in the compensatory strategies employed may create a lower correlation on the group level between gait speed and postural sway measures during the more challenging SI conditions. Lastly, it is possible that this relationship is influenced by how gait speed is assessed. Namely, all of the gait tests were performed in lab environments under optimal conditions, with minimal-to-no sensory disturbance. This may have produced a better gait speed than in real life, potentially leading to associations that did not follow a clear increasing trend between the gait speed and more challenging SI conditions [74,75].

Another interesting finding of the current study was that while there was no association between gait speed and sway velocity during EOS, a significant relationship was found between gait speed and sway velocity in ECS. Similarly to our study, Ganz et al. [76] also used a dynamic sway measurement. However, their results differed from ours as they reported a significant association between gait speed and center of mass (CoM) acceleration during EOS and a non-significant relationship between gait speed and CoM acceleration during ECS [76]. The discrepancy between the studies could be explained by various factors such as the differences in population characteristics and the outcome measurements for evaluating SI. For instance, the participants in the current study were, on average, six years younger and were community dwellers whereas the participants in Ganz et al. [76]’s study were residents of continuous care facilities. It is reasonable to assume that an advancing age could create a greater susceptibility to SI, resulting in differences in the dynamic sway measures during the conditions.

The most striking finding of this meta-analysis was the refutation of our second hypothesis, which stated that there are significant associations between gait speed and sensory quotients. This may be interpreted to mean that sensory reweighting during postural control in healthy older adults was not linked to their gait speed. Several factors might explain this observation. First of all, the studies included in this meta-analysis consisted of “healthy” older adults with no comorbidities that would affect their sensory systems, balance, and gait. Therefore, it is reasonable to assume that these older adults did not rely yet on one specific sensory system (such as the visual system) as their senses and SI processes remained (largely) intact [77]. Secondly, according to Pinto et al. [78], sensory impairment in a single (of five) sensory system was not associated with gait speed. Instead, a global sensory impairment was indeed linked to a decline in speed [78]. This may be the reason that there were no associations between gait speed and one’s dependence on vision and proprioception in the current study. Another reason could be the compensatory strategies employed by the older adults. Earlier studies suggested that SI allows for compensation for the inadequacy of particular sensory systems in healthy older adults [79]. Therefore, the lack of (reliable) information from one of the sensory systems during the testing, for instance the visual system, could be compensated by reweighting the sensory information or increasing the cortical activity for SI [77,79,80]. This could also explain the non-significant association between gait speed and one’s reliance on vision and proprioception in healthy older adults.

Another potential explanation could be the heterogeneity in the composition of the original study populations with respect to fallers. The population analyzed in this review represented a mixture of fallers and non-fallers, with non-fallers constituting the majority. We believe that a possible significant association between gait speed and quotients may be diluted by this mixing. The limited amount of studies in the literature that did discriminate indeed found that fallers rely on their visual or somatosensory systems more compared to non-fallers and young adults [22,28], which supports our notion. Furthermore, it has been established that falling older adults display pronounced changes in body sway under sensory challenging conditions, the RQ, and gait speed compared to non-fallers [32,81,82]. Therefore, if the population in the current study consisted entirely of fallers, the association could have been more pronounced. This finding highlights the importance of accounting for fall status in future studies to better understand the interplay between gait parameters and SI.

Similarly, gait speed was also not linked to the RQ analyzed as sway velocity. The findings may be attributed to how the sway velocity was interpreted. For instance, Souza-Junior et al. [33] described the RQ in sway velocity as “the percentage increase in CoP oscillation velocity from EO to EC condition”. Thus, higher RQ indicated better adaptation to the task and better SI [33]. However, as evident from the negative correlation between gait speed and the RQ in the other studies included in MA 1.6 [52,54], the interpretation of sway velocity was not similar to that in the study by Souza-Junior et al. [33]. Given the small sample size in MA 1.6, it is possible that the non-significant association was a result of a different interpretation of sway velocity. Furthermore, the moderator analysis indicated that the percentage of female participants in the studies may have influenced the effect sizes in MA 1.6. However, this finding should be interpreted cautiously as there were only four studies included in the meta-analysis.

It is, of course, still possible that SI is associated with gait parameters other than gait speed. A study by Mahoney and Verghese [27] revealed that multisensory (visual–somatosensory) integration was significantly associated with pace (a factor that included gait speed, stride length, and the double-support phase) and stride length variability. Additionally, studies investigating the relationship between gait variability or smoothness and body sway in challenging sensory conditions have reported positive correlations [54,76]. Although they did not specifically calculate the quotients, it is reasonable to conclude that the SI could be associated with other parameters of gait. This, however, could not be assessed in the current meta-analysis given the lack of studies in this field.

Lastly, the SI processes in the original studies included in this meta-analysis were evaluated during standing (i.e., static tasks) whereas gait inherently involves dynamic movement. It is reasonable to assume that the SI requirements for static and dynamic tasks differ significantly as dynamic tasks may impose greater demands on the integration of sensory inputs to maintain balance and coordination. This distinction may further explain the non-significant associations observed in this study. To address this gap, we emphasize the importance of developing and utilizing tests specifically designed to evaluate SI during dynamic tasks. For instance, dynamic adaptations of tests like the CTSIB or SOT, such as Locomotor SOT, could provide valuable insights into SI processes during movement [83]. We strongly recommend that future research focuses on such evaluations to better understand the complex relationship between gait parameters and SI under dynamic conditions.

This systematic review and meta-analysis has some limitations. The main limitation of our study was the exclusive focus on static SI assessments. While gait is inherently a dynamic task, we chose to limit our scope due to the considerable methodological heterogeneity across studies evaluating the contribution of sensory systems to dynamic conditions. These studies often employed diverse sensory-challenge protocols, sometimes combining multiple modalities or focusing exclusively on one, which precluded reliable cross-study comparison or meta-analytic synthesis. Focusing solely on SI during static conditions allowed us to examine the gait speed and SI relationship within a more methodologically consistent and comprehensive framework, which strengthened the internal validity and comparability of the findings across studies. Nevertheless, we acknowledge that residual variability stemming from differences in measurement instruments may still have influenced comparability to some extent. Additionally, due to the unavailability of raw data, we were unable to adjust for potential differences between Pearson and Spearman correlation coefficients, which may represent a minor source of bias. Secondly, the risk of bias in the included studies was high, primarily due to data being obtained through author contact, which may have affected the reliability of our findings. Lastly, the exclusion of clinical populations with known disorders (e.g., sensory or neurological) may limit the generalizability of our findings to the broader older adult population. A major strength of this study was that it represented the first systematic review and meta-analysis to comprehensively examine the relationship between gait speed and SI in healthy older adults. By grouping data according to distinct SI conditions, we were able to detect subtle but significant associations between gait speed and sensory conditions, relationships that may be overlooked in individual studies due to small sample sizes or methodological variability. Another strength lay in the inclusion of sensory quotients, which allowed for a more nuanced understanding of sensory system reliance in postural control and its potential links with gait. This added a unique dimension to our analysis that had been rarely explored in the previous literature.

## 5. Conclusions

Significant, yet small, negative associations were found between gait speed and postural sway area under sensory-challenging conditions. However, no significant associations were observed between gait speed and sensory quotients. This divergence highlights a partial relationship between gait speed and SI in healthy older adults. This partial link suggests that healthy older adults may preserve sensory reweighting strategies during quiet standing, but subtle instabilities under challenging conditions could signal early postural control changes preceding gait decline or fall risk. These findings reiterate the value of incorporating SI assessments into clinical screenings to identify early signs of mobility decline and guide fall prevention strategies.

The lack of associations between gait speed and sensory quotients could be attributed to several factors, such as heterogeneity in population characteristics, fall status, outcome measurements, and their interpretations. Furthermore, it may also be partly explained by the absence of SI measurements in dynamic contexts since SI assessments in static conditions may not adequately reflect the demands associated with gait. The findings support the need for dynamic, comprehensive SI evaluations and highlight that future research is needed to develop such assessments.

## Figures and Tables

**Table 1:** List of meta-analyses performed in this study.

Number of the Meta-Analysis	Condition/Quotient	Outcome Measurement
Meta-analysis 1.1	Eyes open/stable surface	Sway area
Meta-analysis 1.2		Sway velocity
Meta-analysis 1.3	Eyes closed/stable surface	Sway area
Meta-analysis 1.4		Sway velocity
Meta-analysis 1.5	Romberg Quotient	Sway area
Meta-analysis 1.6		Sway velocity
Meta-analysis 2.1	Eyes open/complaint surface	Sway area
Meta-analysis 2.2	Eyes closed/complaint surface	Sway area
Meta-analysis 2.3	Proprioception Quotient	Sway area

**Table 2:** Characteristics of the eligible studies.

Author (Year)	Characteristics of Participants	Sensory Integration Conditions and Foot Position	Gait Test	Outcome Measurement(s)	Findings on Sensory Integration conditions	Findings on Quotients	Meta-Analysis
Espinoza-Araneda et al. (2022) [60]	Age: 69.93 ± 4.95 (61–80) F/M: 38/33 BMI: 29.04 ± 3.74 Fallers: NR	EOS ECS EOC ECC Foot position: NR	Unstandardized test (walking for 9 m)	95% confidence ellipse area	**Significant correlations:** Gait speed x EOS: r: −0.253, *p*: 0.034 Gait speed x ECS: r: −0.332, *p*: 0.005 Gait speed x ECC: r: −0.238, *p*: 0.046 Foot clearance x EOS: r:0.237, *p*: 0.047 Foot clearance x ECS: r:0.328, *p*: 0.005 Foot clearance x ECC: r:0.251, *p*: 0.035 Cycle duration x EOS: r:0.238, *p*: 0.046 Cycle duration x ECS: r:0.309, *p*: 0.009	**No significant correlation** between gait speed x RQ and gait speed x PQ **No significant correlation** between foot clearance x RQ and foot clearance x PQ **No significant correlation** between cycle duration x RQ and foot clearance x PQ **No significant correlation** between stride length x RQ and stride length x PQ	MA 1.1 MA 1.3 MA 1.5 MA 2.1 MA 2.2 MA 2.3
Harro & Garascia (2019) [59]	Age: 67.8 ± 5.1 (60–80) F/M: 24/22 BMI: NR Fallers: No fallers	SOT (EOS, ECC) Foot position: hip-distance	10 MWT	Equilibrium score	NR	**No significant correlation** between gait speed x VQ	
Backlund et al. (2017) [52]	Age: 71 ± 4.0 (NR) F/M: 29/29 BMI: NR Fallers: NR	EOS ECS EOC Foot position: self-selected (EOS, EOC) and feet together (EOS, ECS)	10 MWT	Peak-to-peak Sway range Sway velocity of the trunk	**No significant correlation** between gait speed and conditions (for both outcome measurements)	**No significant correlation** between gait speed x RQ and gait speed x PQ (for both outcome measurements)	MA 1.1 MA 1.2 MA 1.3 MA 1.4 MA 1.5 MA 1.6 MA 2.1 MA 2.3
Lord et al. (1996) [53]	Age: 72.8 ± 6.2 (NR) F/M: 96/0 BMI: NR Fallers: 29 (30.2%; Multiple fallers 11.5%)	EOS ECS EOC ECC Foot position: NR	Unstandardized test (Gait data collected for 20 steps)	Sway area of the trunk/CoM	**Significant correlations:** Gait speed x EOC: r: −0.21, *p* < 0.01 Gait speed x ECC: r: −0.17, *p* < 0.05	**No significant correlation** between gait speed x RQ and gait speed x PQ	MA 1.1 MA 1.3 MA 1.5 MA 2.1 MA 2.2 MA 2.3
Labata-Lezaun et al. (2022) [61]	Age: 73.7 ± 7.44 (62–93) F/M: 21/31 BMI: 28.3 ± 4.12 Fallers: NR	EOS ECS Foot position: at a 30° angle, heels 2 cm apart	4 MWT	95% confidence ellipse area	**Significant correlations:** Gait speed x EOS: r: −0.31, *p*: 0.025	**No significant correlation** between gait speed x RQ	MA 1.1 MA 1.3 MA 1.5
Camicioli et al. (1997) [34]	Age: 83.2 (66–102) F/M: 24/24 BMI: NR Fallers: 19 (21.6%)	SOT (EOC, ECC) Foot position: hip-distance	Unstandardized test (walking for 9 m)	Equilibrium score	**No significant correlation** between gait speed and conditions	Quotients are NR	
Souza-Junior et al. (2022) [33]	Age: 69.3 ± 5.9 (NR) F/M: 60/0 BMI: 26.6 ± 4.4 Fallers: NR	EOS ECS Foot position: at a 30° angle, heels 6 cm apart	3 MWT	95% confidence ellipse area Mean CoP velocity	**No significant correlation** between gait speed and conditions (for both outcome measurements)	**Significant correlations:** gait speed x RQ: r:0.339, *p*: 0.001 (mean velocity)	MA 1.1 MA 1.2 MA 1.3 MA 1.4 MA 1.5 MA 1.6
Scaglioni-Solano & Aragon-Vargas (2015) ^¥^ [54]	Age: 70.6 ± 5.7 (NR) F/M: 74/26 BMI: 27.0 ± 4.2 Fallers: Included fallers	EOS ECS EOC ECC Foot position: NR	10 MWT	Mean CoP velocity	**No significant correlation** between gait speed and conditions	**No significant correlation** between gait speed x RQ and gait speed x PQ	MA 1.2 MA 1.4 MA 1.6
Li et al. (2010) [55]	Age: 76.2 (NR) F/M: 13/7 BMI: NR Fallers: NR	SOT (EOS, EOS with sway-referenced visual surroundings, EOC) Foot position: hip-distance	6 min Walk Test	Equilibrium score	**No significant correlation** between gait speed and conditions	**No significant correlation** between gait speed x PQ	
Hupfeld et al. (2021) [56]	Age: 72.8 (NR) F/M: 11/12 BMI: 26.0 ± 3.9 Fallers: NR	mCTSIB (EOS, ECS, EOC, ECC) Foot position: NR	4 min Walk Test	95% confidence ellipse area (CoM)	**No significant correlation** between gait speed and conditions	**No significant correlation** between gait speed x RQ and gait speed x PQ	MA 1.1 MA 1.3 MA 1.5 MA 2.1 MA 2.2 MA 2.3
White et al. (2021) [57]	Age: 75.4 ± 5.3 (NR) F/M: 31/25 BMI: NR Fallers: 20% single or multiple fallers	EOS ECS EOC ECC Foot position: NR	Unstandardized test (walking for 23 m)	Trace Length (mm)	**Significant correlations:** Gait speed x EOS: r: −0.377, *p*: 0.004 Gait speed x ECS: r: −0.298, *p*: 0.026 Gait speed x EOC: r: −0.330, *p*: 0.013	**No significant correlation** between gait speed x RQ and gait speed x PQ	MA 1.1 MA 1.3 MA 1.5 MA 2.1 MA 2.2 MA 2.3
Si et al. (2024) ^θ^ [37]	Age: 67.4 (NR) F/M: 32/23 BMI: 23.7 Fallers: NR	EOS ECS Foot position: self-selected and feet together in both conditions	Unstandardized test (walking for 5.2 m)	Sway Velocity Index 95% confidence ellipse area	***Sway Velocity Index*** **No significant correlations** between gait speed and conditions **in self-selected stance** **Significant correlations in narrow stance:** Gait speed x ECS: r: −0.350, *p*: 0.009 ***95% confidence ellipse area *** **Significant correlations in self-selected stance:** Gait speed x ECS: r: −0.273, *p*: 0.044 **Significant correlations in narrow stance:** Gait speed x ECS: r: −0.387, *p*: 0.004 ***Sway Velocity Index*** **No significant correlations** between cadence and conditions **in self-selected stance** **Significant correlations in narrow stance:** Cadence x ECS: r: −0.281, *p*: 0.038 ***95% confidence ellipse area*** **No significant correlations** between cadence and conditions **in self-selected stance** **Significant correlations in narrow stance:** Cadence x ECS: r: −0.279, *p*: 0.039	Quotients are NR	MA 1.3 MA 1.4
Varjan et al. (2024) [58]	Age: 72.7 ± 4.4 (65–75) F/M: 34/0 BMI: NR Fallers: NR	EOS ECS Foot position: hip-width apart, toes pointing outwards	10 MWT	Mean CoP velocity	**No significant correlations** between gait speed and conditions	**No significant correlation** between gait speed x RQ	MA 1.2 MA 1.4 MA 1.6

¥: This study originally included 122 older adults; however, only the data for 100 participants were shared with the authors. θ: This study only reported statistically significant correlations and therefore, it was not possible to include it in every possible meta-analysis. EOS: Eyes-open stable surface. ECS: Eyes-closed stable surface. EOC: Eyes-open compliant surface. ECC: Eyes-closed compliant surface. RQ: Romberg Quotient. PQ: Proprioception Quotient. NR: Not reported. SOT: Sensory Organization Test. mCTSIB: Modified Clinical Test of Sensory Interaction in Balance. 10 MWT: 10-Meter Walk Test. CoM: Center of Mass. NA: Not applicable. VQ: Vestibular Quotient. 4 MWT: 4-Meter Walk Test. MA: Meta-analysis.

**Table 3:** Risk of bias in the studies included in the meta-analysis.

Author (Year)	Study Participation	Study Attrition	Prognostic Factor Measurement	Outcome Measurement	Study Confounding	Statistical Analysis and Reporting	Overall
Espinoza-Araneda et al. (2022) [60]	High	Low	Moderate	Moderate	High	High	**High**
Harro & Garascia (2019) [59]	High	Low	Low	Low	High	High	**High**
Backlund et al. (2017) [52]	High	Low	Moderate	Low	High	High	**High**
Lord et al. (1996) [53]	High	High	High	Moderate	High	High	**High**
Labata-Lezaun et al. (2022) [61]	High	Low	Moderate	Low	High	High	**High**
Camicioli et al. (1997) [34]	High	Low	Low	Moderate	Moderate	Moderate	**High**
Souza-Junior et al. (2022) [33]	High	High	Low	Low	Moderate	Low	**High**
Scaglioni-Solano & Aragon-Vargas (2015) [54]	High	High	Low	Low	High	High	**High**
Li et al. (2010) [55]	High	High	Moderate	Low	High	High	**High**
Hupfeld et al. (2021) [56]	High	High	Moderate	Low	High	High	**High**
White et al. (2021) [57]	High	High	High	Moderate	High	High	**High**
Si et al. (2024) [37]	High	Low	Low	Low	High	Low	**High**
Varjan et al. (2024) [58]	High	Low	Low	Low	High	High	**High**

**Table 4:** Findings of individual meta-analyses.

Meta-Analysis	Condition/Quotient and Outcome Measurement	*n*	*n*	Pooled Effect Size	95% CI	*p*-Value	I^2^
Meta-analysis 1.1	Gait speed x EOS (sway area) 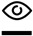	7	416	−0.235	−0.340–(−0.13)	**<0.001**	0%
Meta-analysis 1.2	Gait speed x EOS (sway velocity) 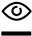	4	252	−0.056	−0.213–0.101	0.486	31.6%
Meta-analysis 1.3	Gait speed x ECS (sway area) 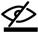	9	526	−0.201	−0.306–(−0.102)	**<0.001**	24.6%
Meta-analysis 1.4	Gait speed x ECS (sway velocity) 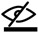	5	307	−0.149	−0.266–(−0.37)	**0.01**	0%
Meta-analysis 1.5	Gait speed x RQ (sway area) 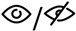	7	416	0.034	−0.065–0.133	0.499	0%
Meta-analysis 1.6	Gait speed x RQ (sway velocity) 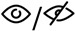	4	252	0.008	−0.240–0.256	0.949	72.0%
Meta-analysis 2.1	Gait speed x EOC (sway area) 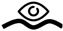	5	304	−0.198	−0.316–(−0.086)	**<0.001**	0%
Meta-analysis 2.2	Gait speed x ECC (sway area) 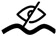	4	246	−0.186	−0.316–(−0.060)	**0.004**	0%
Meta-analysis 2.3	Gait speed x PQ (sway area) 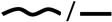	5	304	−0.068	−0.183–0.047	0.249	0%

Bold numbers indicate significance (*p* < 0.05). EOS: Eyes-open stable surface. ECS: Eyes-closed stable surface. EOC: Eyes-open compliant surface. ECC: Eyes-closed compliant surface. RQ: Romberg Quotient. PQ: Proprioception Quotient.

## Data Availability

The data utilized in this systematic review were obtained directly from the authors of the original studies upon request and were provided with the explicit understanding that they would be used solely for the purposes of this review and not be shared further. As such, the datasets are not publicly available and cannot be shared by the authors of the present study.

## Supplementary Materials

The following supporting information can be downloaded at: https://www.mdpi.com/article/10.3390/jcm14134545/s1, Supplementary Material S1—Search Strategy based on PICO. Supplementary Material S2—Supplementary Figure S1. Meta-regression analysis showing the distribution of female gender as a moderator.
