# Supplementary material for: Does Sensory Integration Influence Gait Parameters in Healthy Older Adults? Insights from a Systematic Review with Meta-Analysis
Source: J Clin Med. 2025 Jun 26;14(13):4545. doi: 10.3390/jcm14134545 (PMC12250018; doi:10.3390/jcm14134545)
Supplement: Supplementary file 1 [file jcm-14-04545-s001.zip › Supplementary Material A- Search strategy.pdf]

Database Search

Appendix 1. Search Strategy based on PICO

**PICO**	**Explanation**	**Keywords**
**P**- Population	Healthy community-dwelling older adults (>60 years old)	("old*" OR "aged" OR "elder*")
**I**- Intervention/ Prognostic Factor	Sensory interaction/integration	(("posturograph*" OR "sensory organization" OR "sensory interaction" OR "sensory reweighting" OR "sensory re-weighting" OR "sensory re-weighing" OR "center of pressure" OR "centre of pressure" OR "center of mass" OR "centre of mass" OR "stabilometr*" OR "SwayStar" OR "Sway Star" OR "postural sway" OR "sway" OR "postural balance" OR "postural control" OR "postural stability") AND ("eyes open" OR "eyes closed" OR "surface" OR "sens*" OR "propriocept*" OR "vestibul*" OR "galvanic" OR "mastoid vibration")
**C**- Comparison	Not applicable.	
**O**- Outcome	Gait characteristics	("gait" OR "walk*" OR "locomot*")
