# Supplementary material for: Does Sensory Integration Influence Gait Parameters in Healthy Older Adults? Insights from a Systematic Review with Meta-Analysis
Source: J Clin Med. 2025 Jun 26;14(13):4545. doi: 10.3390/jcm14134545 (PMC12250018; doi:10.3390/jcm14134545)
Supplement: Supplementary file 1 [file jcm-14-04545-s001.zip › Supplementary Material B- meta-regression.pdf]

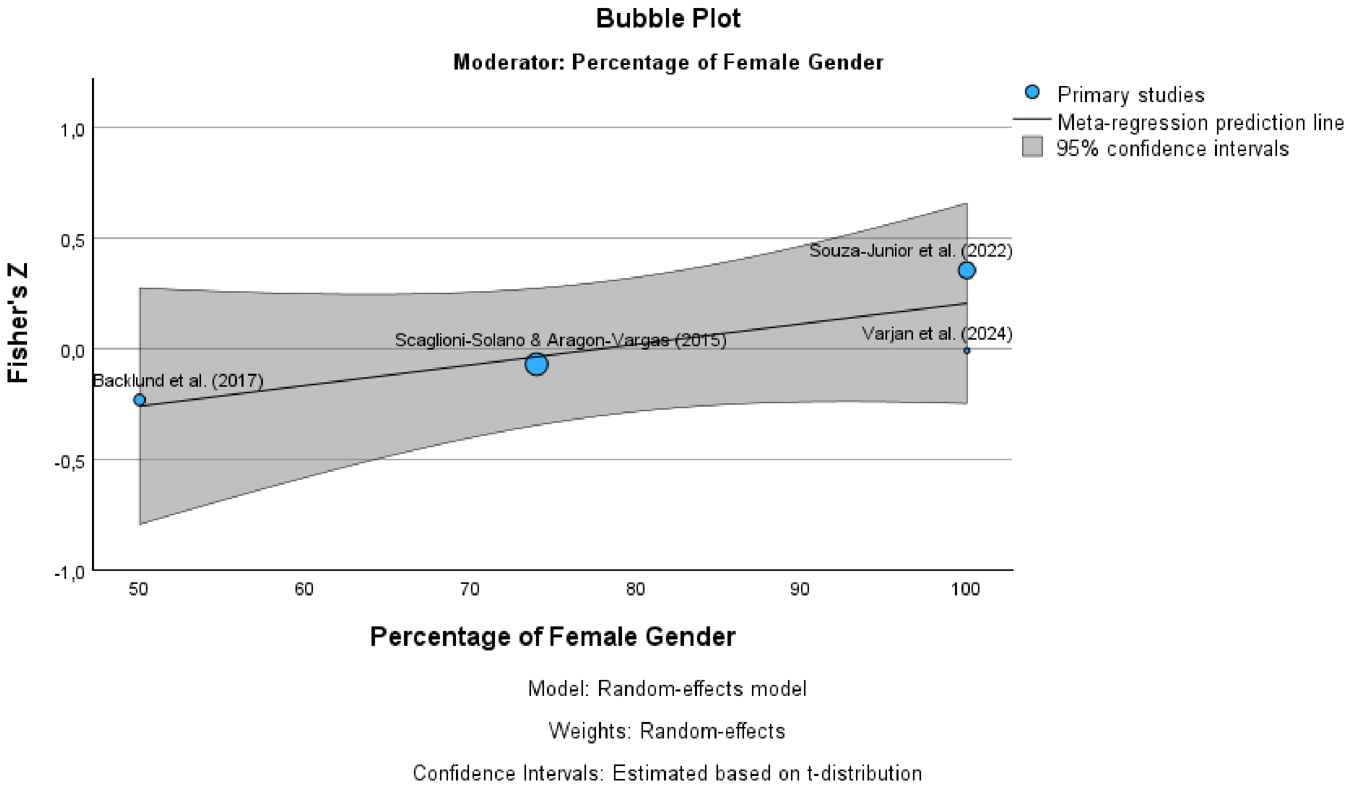

Supplementary Figure 1. Meta-regression analysis showing the distribution of female gender as a moderator.
